# P314: Human Immunodeficiency Virus (HIV) and Hepatitis B Virus (HBV) infection prevention following occupational exposure among staff at regional referral hospital in W. Kenya

**DOI:** 10.1186/2047-2994-2-S1-P314

**Published:** 2013-06-20

**Authors:** BK Burmen, DK Bii, J Obunga, P Olilo

**Affiliations:** 1 HIV and TB Implementation Science and Services , Kenya Medical Research Institute/Centers for Disease Control Research and Public Health Collaboration, Kisumu, Kenya; 2 Clinical, jaramogi oginga odinga teaching and referral hospital, Kisumu, Kenya

## Introduction

Post exposure prophylaxis (PEP) with antiretroviral therapy (ART) aids in preventing HIV infection from accidental or occupational exposure. Hospital staff should be vaccinated against HBV to protect them in the event of accidental exposure to blood and body fluids.

## Objectives

To ascertain adherence and completion of PEP among staff at a regional referral hospital in W. Kenya.

## Methods

We reviewed staff PEP registers at a Regional Referral hospital in Western Kenya between January 2011 and December 2012. National PEP guidelines recommend ART initiation within 72 hours of exposure, use of ART for28 days post-exposure and HIV re-testing at1.5, 3 and 6 months following ART initiation. A high-risk exposure occurs when a source is HIV-positive with injury on a mucosal membrane by needleprick, cut or splash. The risk of HBV infection increases if the exposed is not HBV-vaccinated and source of exposure is HBV-positive. Chi Square statistics were used to determine factors associated with PEP completion.

## Results

Of 52 hospital staff who initiated PEP; 33 were health workers (63%), 3 support staff (6%), 14 students (27%), and 2 unknown cadres (4%). Half were female (n=27); 29 from in-patient units (56%) and 30 had high-risk exposures (58%).Of the staff with documented timing of both HIV exposure and ART initiation (n=47), ART was initiated within the recommended time. Half of all staff (n=27) completed PEP. Reasons for non-completion were side effects (n=2), referral (n=1) and unknown (n=23). PEP completion did not vary by gender (p=0.78), exposure-type (p=1.0) or exposure-unit (p=0.75).At 1.5, 3, and 6 months after ART initiation, HIV re-testing rates were 96%, 25%, 17%and negativity rates were 100%, 100%,75%, respectively.Only17% (n=9) of staff were HBV-vaccinated;94%(n=49) of sources had unknown HBV status. No intervention was documented HBV prevention.

## Conclusion

Despite timely PEP initiation, low rates of completion negate its intended benefits. Universal precaution-practices and PEP completion should be enforced; so should HBV staff-vaccination and testing for both staff and sources of exposure to tackle the low rates of HBV testing and vaccination.

## Disclosure of interest

None declared

